# Antiretroviral therapy initiation within seven days of enrolment: outcomes and time to undetectable viral load among children at an urban HIV clinic in Uganda

**DOI:** 10.1186/s12879-017-2550-2

**Published:** 2017-06-19

**Authors:** Rogers Ssebunya, Rhoda K. Wanyenze, Heather Lukolyo, Milton Mutto, Grace Kisitu, Pauline Amuge, Albert Maganda, Adeodata Kekitiinwa

**Affiliations:** 1 Baylor College of Medicine Children’s Foundation, Mulago Hospital Complex, P.O. Box 72052, Kampala, Uganda; 2 School of Public Health, Makerere University College of Health Sciences, P.O. Box 7072, Kampala, Uganda

**Keywords:** HIV, Undetectable viral load, Antiretroviral therapy, Outcomes, Timing

## Abstract

**Background:**

Viral suppression is a critical indicator of HIV treatment success. In the era of test-and-start, little is known about treatment outcomes and time to undetectable viral loads. This study compares treatment outcomes, median times to achieve undetectable viral loads and its predictors under different antiretroviral (ART) treatment initiation schedules (i.e. within seven days of enrolment or later).

**Methods:**

A retrospective cohort of 367 patients <18 years who enrolled in care between January 2010 and December 2015 with a baseline viral load of >5000 copies/ml were followed up for 60 months. Undetectable viral load measurements were based on both Roche (<20copies/ml) and Abbot (<75copies/ml). Clinical treatment outcomes were compared using chi-squared test. Survival experiences between the two cohorts were assessed through incidence rates and Kaplan Meier curves. A cox model with competing risks was used to assess predictors for time to undetectable viral load.

**Results:**

Of the 367 patients, 180 (49.1%) initiated ART within seven days from enrolment, 192 (52.3%) attained undetectable viral load of which 133 (69.3%) were children below six years and 101 (52.6%) were females. Among those who initiated ART within seven days 15 (8.3%) died and 6 (3.3%) were lost to follow-up compared to 27 (14.4%) and 16 (8.6%) respectively in the later initiators. The median time to undetectable viral load was 24.9 months (95% CI: 19.7, 28.5) among early ART initiators and 38.5 months (95% CI: 31.1, 44.5) among those initiating beyond seven days. There was a significant difference in failure estimates between those initiating within seven and those that deferred (log rank, *p* = 0.001). Significant predictors for time to undetectable viral load were; starting ART within seven days (SHR = 2.02, 95% CI: 1.24, 3.28), baseline WHO stage I or II (SHR = 1.59, 95% CI: 1.06, 2.28), inconsistent adherence on three consecutive clinic visits (SHR = 0.44, 95% CI: 0.28, 0.67), and baseline weight (SRH = 1.04, 95% CI: 1.01, 1.07).

**Conclusion:**

Prompt initiation of ART within the first week of enrolment is associated with better treatment outcomes. Early timing, baseline WHO clinical stage and adherence rates should be major considerations while managing HIV among children.

## Background

Over the past decade, the world has achieved unprecedented milestones in the fight against the HIV epidemic highlighted by reduced incidence and mortality rates [1–3]. Some of the benchmarks to these successes encompass combination prevention strategies including treatment as prevention [4]. Antiretroviral therapy (ART), especially when initiated early after HIV diagnosis, is associated with better prognosis and quality of life [5–8] as well as reduced HIV transmission rates [9]. In efforts to reach the goal of ending AIDS by 2030, focus on the sub-Saharan African countries where 70% of the people living with HIV globally dwell [10] is vital.

In 2013, the World Health Organization (WHO) recommended that all children less than five years of age be initiated on ART irrespective of CD4 count or clinical staging [11]. Recently the START study documented the benefits of early ART initiation for all individuals newly diagnosed with HIV [6] and Uganda is currently implementing the “test and start” approach for early initiation of ART among children below 15 years following the 2014 national treatment guidelines. Amidst the evidence on the feasibility of starting ART on the first day of HIV diagnosis [12] and the critical role on treatment outcomes, the current guidelines do not provide clear direction in terms of the actual timing of treatment initiation. In practice, it may take a few days for treatment initiation to be effected since many HIV testing facilities may not have capabilities to initiate treatment and newly diagnosed HIV infected individuals have to be referred elsewhere for ART. It is not known if treatment outcomes of those who start within a few days (e.g. as early as seven days of diagnosis) are better than those that defer treatment.

High levels of HIV viremia is one of the greatest risk factors in HIV transmission [13, 14], making viral suppression a cornerstone in improving treatment outcomes and the quality of life. Results from the Partner study show a zero risk of HIV transmission after condomless sex from an HIV positive individual’s whose viral load is <200 copies/ml [15]. It is therefore critical to monitor viral load levels of all individuals initiated on ART, among other outcomes. A number of studies have documented evidence related to immunological recovery and viral suppression and their related covariates. There is evidence of better immunological and virological responses among older individuals than younger ones [16–18]. Baseline viral loads, CD4 count and regimen have all been linked to immunological response among patients in care [19, 20]. Higher baseline viral loads were significantly related to failure to achieve undetectable viral load in a US based cohort analysis [21] and studies elsewhere [22, 23]. Protease (PI) based regimens have had mixed findings with some indicating better response [16, 24] and others were contradictory [16, 25] or did not show any differences between the PI and NVP based regimens [20]. Some studies have indicated that females are more likely to attain undetectable viral load [26] while in others there were no gender differences. ART adherence rates have also been linked to better treatment outcomes [27–29]. However, few studies have documented the role of baseline weight, haemoglobin level and nutritional status on the time to undetectable viral load [30] and early initiation (within a week of diagnosis) compared to later initiation on undetectable viral load. This study therefore aimed to compare the treatment outcomes among patients who start treatment within seven days and after seven days of enrolment in care. We further intended to describe survival experiences, and predictors of time to undetectable viral load between the two groups.

## Methods

### Study setting

Baylor College of Medicine Children’s Foundation-Uganda (Baylor-Uganda) is a private-not-for-profit organisation that runs a centre of excellence HIV clinic within the Mulago National Referral and Teaching Hospital campus in Kampala. It is an HIV clinic that has been in existence since 2003 that provides family-centred care to more than 7000 children and adolescents and their families. Focus on the child’s health and their caretakers is central in this family-centered approach. All patients recruited at this clinic receive care and treatment free of charge, based on the national guidelines and WHO provisions.

### Study design and population

This was a retrospective cohort design involving 383 participants receiving care at an HIV clinic at the Baylor-Uganda centre of excellence. Patients enrolled in care from 1st January 2010 to 30th November 2015 both active and inactive (dead, lost to follow-up, transferred out) were followed up for a maximum of five years. Patients’ records with two or more viral load measurements were retrieved from an Electronic Medical records (EMR) database. From the total sampled clients (*n* = 4336), only clients’ bearing a baseline viral load (*n* = 418) were considered for this study. Baseline viral load values were considered as any viral load result obtained between three months before to three months after ART initiation. Clients with at least two viral load results with one being the baseline of >5000 copies/ml were selected. The 2014 policy in Uganda recommended viral load testing six months after ART initiation however VL monitoring at this centre of excellence started earlier than 2014 with support from an international organisation.

### Data collection procedures

Data were extracted on; socio-demographic characteristics, treatment regimens for HIV and medications used to treat opportunistic infections, adherence measurements, treatment outcomes (clinical staging, immunological and virological) and selected laboratory tests done. Individual characteristics abstracted included; date of enrolment, date of ART initiation, baseline regimen, current chart status (i.e. alive and in care, dead, lost to follow-up or transferred out), history of current or past tuberculosis, and nutritional status computation based on MUAC, weight and age) Laboratory tests abstracted included; CD4 count (absolute and percent), viral load, haemoglobin and creatinine. Data was cleaned and exported to Stata statistical software version 13.0 for analysis.

### Variables measurements

The outcome of interest in this study was time to undetectable viral load (defined as time from baseline viral load to viral load less than 75 copies/ml) after ART initiation among all those whose baseline viral load was >5000 copies/ml. Viral loads measurements at this site were based on two criteria; Abbot Machine (<75 copies/ml) and Roche machine (<25 copies/ml). Timing of ART initiation, categorized as within seven days and beyond seven days from the date of enrolment in care, was an independent variable. The seven-day cut-off period was guided by the recommendations in the 2016 HTC policy and treatment guidelines for Uganda [31]. Other independent variables included; age, sex, baseline WHO clinical stage, ART regimen, viral load count, CD4 count, nutrition status. Age was categorised as 0–5, 6–15, and >15 years. Baseline regimen initiated on was categorised into; Nevirapine (NVP)-based regimen, Efavirenz (EFV)-based regimen, protease inhibitor (PI)-based regimen, and non-nucleoside reverse transcriptase inhibitor (NNRTI) regimens. Other variables categorised included; adherence rates; ≥95% coded as good, 85–94% coded as fair, and below 85% coded as poor based on pill count or self-reported adherence during the clinic visit. Inconsistent adherence in this study referred to patients who never had good adherence on 3 consecutive clinic visits.

### Statistical analysis

Comparison of proportional and mean differences in the viral load were analysed using a chi-squared test for categorical variables and an independent two sample t-test for continuous variables. Clinical outcomes among those who initiated within and beyond seven days were analysed using a chi-squared test while comparison of rate of occurrence of undetectable viral load within the strata of age, baseline CD4 count and timing of ART initiation were analysed as incidence rates. Survival experience between early ART initiators and later initiators were analysed and presented using Kaplan Meier curves and differences within assessed using a log rank test. Predictors for time to undetectable viral load were analysed using a cox model with competing risks. Model parsimony, diagnostics on both goodness of fit and violation of proportional hazard (PH) assumptions were also carried out before the final model was concluded. Survival analysis was sought to be the most appropriate analysis in this study because analysis of outcome of interest is best on the exposure time of each participant in the study. We report our findings in line with the recommended Strengthening of Observational Studies in Epidemiology (STROBE) statement guidelines [32].

## Results

### Baseline characteristics of study participants

Demographic and baseline clinical and laboratory characteristics of study participants are summarized in Table 1. Study participants ranged in age from 6 weeks to 18 years, with the majority (73.3%) falling below six years. At enrolment, most of the participants were staged in WHO clinical 3 or 4 (62.9%), had an absolute CD4 count above or equal to 500 cells/μl (70.3%) and were within the normal nutrition limits (49.6%).

**Table 1:** Baseline characteristics of study participants

Variable, *N* = 367		N	Percent (%)
Age	0–5	269	73.30
	6–15	83	22.62
	15–18	15	4.09
Sex	Male	175	47.68
	Female	192	52.32
WHO clinical stage	I & II	136	37.06
	III & IV	231	62.94
Nutritional status^a^ (*n* = 335)	Within normal limits	166	49.55
	Mild malnutrition	26	7.76
	Moderate malnutrition	61	18.21
	Severe malnutrition	82	24.48
CD4 absolute count	<500 cells/ml	109	29.70
	> = 500 cells/ml	258	70.30
Baseline viral load (copies/ml)^b^	5000–100,000	102	27.79
	100,000–500,000	107	29.16
	500,000–1,000,000	52	14.17
	≥1,000,000	106	28.88
Haemoglobin level, gm/dl (*n* = 271)	0–8	42	15.50
	8–16	229	84.50

^a^Baseline status based on the Z-score criteria

^b^Measurements based on either Roche or Abbot

It was observed that baseline weight and consecutive good ART adherence rates were associated with undetectable viral load as illustrated in Table 2. Individuals who attained undetectable viral loads within the follow-up period were slightly heavier; mean weight = 14.4 (SD-14.0), had higher baseline haemoglobin level, mean 10.0 g/dl (SD-1.9). In both cohorts, Nevirapine and Protease inhibitor-based ART regimens were prescribed at start of treatment. It was observed that a slightly higher proportion started on a triple reverse transcriptase inhibitors (RTIs) never attained undetectable viral loads within the 60 months’ follow-up period. Table 3 shows a significant proportion, 173 (92.5%) of children aged 0–5 years were initiated late on ART as well as those within normal and mild malnutrition levels, 109 (58.3%).

**Table 2:** Results showing relationship between baseline characteristics and undetectable viral load among study participants

Variable	Undetectable viral load, *N* = 367		*P* value
	Yes, n (%)	No, n (%)	
Sex			0.908
Male	91 (47.4)	84 (48.0)	
Female	101 (52.6)	91 (52.0)	
Age group			0.163
0–5	133 (69.3)	136 (77.7)	
6–15	49 (25.5)	34 (19.4)	
15–18	10 (5.2)	5 (2.9)	
Timing of ART initiation			0.065
Within 7 days	103 (53.7)	77 (44.0)	
Beyond 7 days	89 (46.3)	98 (56.0)	
Mean baseline weight (SD)	14.4 (14.0)	11.1 (9.4)	0.007*
Baseline creatinine level	28.6 (11.2)	27.6 (12.0)	0.705
Baseline haemoglobin level	10.02 (1.9)	9.57 (1.64)	0.020*
Adherence on ART			0.001*
Good adherence (Pill count based) (≥3 consecutive visits)	38 (19.8)	72 (41.1)	
Inconsistent adherence	154 (80.2)	103 (58.9)	
Baseline Nutrition status			0.745
Normal limits & mild malnutrition	102 (53.1)	90 (51.4)	
Moderate & severe malnutrition	90 (46.9)	85 (48.6)	
TB status			0.067
Positive	15 (7.8)	24 (13.7)	
Negative	177 (92.2)	151 (86.3)	
Baseline ART regimen			0.121
Nevirapine based	66 (34.4)	77 (44.0)	
Efavirenz based	44 (22.9)	28 (16.0)	
Protease Inhibitor based	75 (39.1)	60 (34.3)	
Triple RTI	7 (3.7)	10 (5.7)	
WHO clinical stage			0.089
I & II	79 (41.2)	57 (32.6)	
III & IV	113 (58.8)	118 (67.4)	

*Significance at *p* < 0.05

**Table 3:** Treatment outcomes and baseline characteristics by timing of ART initiation

Variable	Timing of ART		
	Within 7 days, n (%)	Beyond 7 days, n (%)	*p*-value
Sex			0.055
Male	95 (52.8)	80 (42.8)	
Female	85 (47.2)	107 (57.2)	
Age group			0.001*
0–5	96 (53.3)	173 (92.5)	
6–15	76 (42.2)	7 (3.7)	
15–18	8 (4.4)	7 (3.7)	
Adherence on ART			0.012*
Good adherence (≥3 consecutive clinic visits)	65 (36.1)	45 (24.1)	
Inconsistent adherence	115 (63.9)	142 (75.9)	
Baseline Nutrition status			0.020*
Normal limits & mild malnutrition	83 (46.1)	109 (58.3)	
Moderate & severe malnutrition	97 (53.9)	78 (41.7)	
Baseline ART regimen			0.001*
Nevirapine based	45 (25.0)	98 (52.4)	
Efavirenz based	58 (32.2)	14 (7.5)	
Protease Inhibitor based	73 (40.6)	62 (33.2)	
Triple RTI	4 (2.2)	13 (7.0)	
WHO clinical stage			0.001*
I & II	52 (28.9)	84 (44.9)	
III & IV	128 (71.1)	103 (55.1)	
Treatment outcome			
Alive and on treatment	141 (78.3)	123 (65.8)	0.026*
Dead	15 (8.3)	27 (14.4)	
Lost to follow-up	6 (3.3)	16 (8.6)	
Transferred out	10 (10.0)	21 (11.2)	

*Significance at *p* < 0.05

### Treatment outcomes

Time to initiation of ART from enrolment ranged from 0 to 1314 days. Overall individuals who started ART within the first seven days of enrolment had better treatment outcomes as seen in Table 3. Compared to those who initiated ART after seven days, those who started treatment in the first seven days had higher proportions who remained alive and on treatment (78.3% vs. 65.8%), lower proportions who were lost to follow-up (3.3% vs. 8.6%) and fewer mortalities (8.3% vs. 14.4%) (*p* = 0.026).

### Survival experiences

**Fig. 1:**
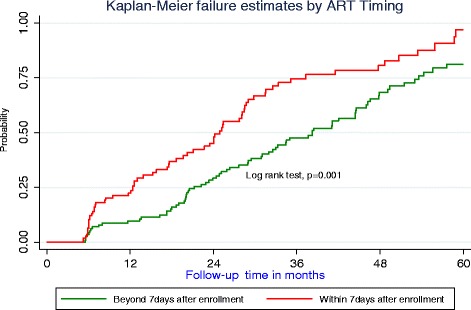
Kaplan Meier curves showing failure experiences of patients initiated on ART within and beyond 7 days of enrolment

Figure 1 shows survival experiences (probability) of undetectable viral load between individuals who initiated ART within and beyond seven days after enrolment. In the first five to six months, both cohorts had similar experiences, until around the seventh month when the survival gradient among those who initiated treatment within seven days rose remarkably compared to those who initiated later. From the 13th month onwards, both cohorts’ survival experiences rose at almost the same gradient till around the 37th month. These survival experiences were statistically significantly different, log rank test, *p* = 0.001. The median time to undetectable viral load for individuals who initiated ART within seven days and that of those beyond seven days were 24.9 months (95% CI: 19.7, 28.5) and 38.3 months (95% CI: 31.1, 44.5), respectively.

Table 4 shows the rate of occurrence of undetectable viral load among the two cohorts. Patients who started treatment within seven days had a rate of 31.6 (95% CI: 25.03, 39.99) as compared to those beyond seven days, rate = 19.9 (95% CI: 15.87, 24.95). Individuals older than 15 years had a 2.3 times higher chance of attaining undetectable viral load as compared to those below six years keeping other age group constant (Unadj.HR = 2.32, 95% CI: 1.17, 4.60). Additionally, individuals who initiated ART within seven days of enrolment had an 88% more chance of attaining undetectable viral loads compared to those who initiated treatment >7 days of enrolment (Unadj.HR = 1.88, 95% CI: 1.35, 2.62).

**Table 4:** Results showing rate of occurrence of undetectable viral load across age groups, baseline CD4 strata and timing of ART initiation

Variable	N	Number of events	Person years per 1000	Rate of undetectable VL (95% CI)	95% CI	HR	95% CI	*P* – value
Age group								
0–5 (ref)	269	105	4.72	22.27	18.39–26.96			
6–15	83	31	1.07	29.03	20.42–41.28	1.35	0.90–2.01	0.144
15–18	15	9	0.20	45.43	23.64–87.32	2.32	1.17–4.60	0.016*
CD4 count (cells/μl)								
< 500 (ref)	109	35	1.43	24.40	17.52–33.99			
> = 500	258	110	4.54	24.19	20.07–29.16	0.93	0.63–1.35	0.688
Timing								
Beyond 7 days (ref)	187	75	3.77	19.90	15.87–24.95			
Within 7 days	180	70	2.21	31.64	25.03–39.99	1.88	1.35–2.62	0.001*

*Significance at *p* < 0.05

### Predictors of undetectable viral load

Table 5 illustrates the significant determinants of time to undetectable viral load among those who initiated ART within and beyond seven days after enrolment. Timing of ART initiation, consecutive adherence rates based on pill count, baseline WHO clinical stage and patient’s baseline weight were significantly associated with time to undetectable viral load. Participant age was not significantly associated with time to undetectable viral load. Patients who were initiated on ART within seven days were 2.02 times higher of attaining undetectable viral loads compared to those who deferred treatment, SHR = 2.02 (95% CI: 1.24, 3.28, *p* = 0.005). Compared to clients who had good adherence(≥95%) on 3 consecutive clinic visits, those who had inconsistent adherence rates to medication within the follow-up period had a 0.44 times the risk of attaining undetectable viral load; SRH = 0.44 (95% CI: 0.28, 0.67, *p* = 0.001). Adherence support from guardians and or parents was crucial for most of the children with good adherence rates of >95%. Additionally patients who were enrolled in care with WHO clinical stage I or II were 1.59 times more likely to achieve undetectable viral load compared to those in clinical stage 3 or 4; SRH = 1.59 (95% CI: 1.06, 2.28, *p* = 0.024). Table 5 also show that for every unit increase in baseline weight of the patient, there was 1.04 times higher chance of attaining undetectable viral load; SRH = 1.04 (95% CI: 1.01, 1.07, *p* = 0.019). On the hand however, the baseline regimens patients initiated on showed no influence on the time to undetectable viral load after controlling for other factors.

**Table 5:** Multivariate analysis results showing predictors for undetectable viral load

Variable	*N* = 367		%	Unadj. HR	95% CI	SHR^a^	95% CI	*P*
Age category	0–5	269	73.30	1.0 (ref)				
	6–15	83	22.62	1.34	0.90–2.03	0.49	0.17–1.38	0.174
	15–18	15	4.09	2.32	0.84–6.40	0.37	0.06–2.15	0.270
ART initiation	Beyond 7 days after enrolment	187	50.95	1.0 (ref)				
	Within 7 days after enrolment	180	49.05	1.88	1.35–2.62	2.02	1.24–3.28	0.005*
Baseline CD4 count (cells/ μl)	<500	109	29.70	1.0 (ref)				
	> = 500	258	70.30	0.92	0.62–1.38	1.36	0.80–2.32	0.252
Adherence to ART	Good adherence on > = 3 consecutive visits	110	29.97	1.0 (ref)				
	Inconsistent adherence	257	70.03	0.67	0.48–0.93	0.44	0.28–0.67	0.001*
ART regimen	NVP based	143	38.96	1.0 (ref)				
	EFV based	72	19.62	2.03	1.17–3.51	1.33	0.68–2.60	0.407
	PI based	135	36.78	1.38	0.97–1.96	1.31	0.87–1.98	0.201
	Triple RTI regimen	17	4.63	0.51	0.18–1.46	0.39	0.09–1.63	0.196
Baseline Viral load (copies/ml)	>100,000	265	72.21	1.0 (ref)				
	<100,000	102	27.79	1.44	1.03–2.03	0.84	0.55–1.27	0.411
Baseline WHO stage	Stage III or IV	231	62.94	1.0 (ref)				
	Stage I or II	136	37.06	1.55	1.11–2.18	1.59	1.06–2.28	0.024*
Baseline weight				1.02	1.01–1.03	1.04	1.01–1.07	0.019*

*Significance at *p* < 0.05

^a^ *SHR* sub hazard ratio

## Discussion

Our results show that ART initiation within the first seven days of enrolment, consistently high adherence rates >95%, lower baseline WHO clinical stage, and weight are significantly associated with achieving undetectable viral load among HIV-1 clients starting treatment for the first time. Similar and contrasting findings elsewhere have been linked to undetectable viral load [6, 16, 22, 23, 26]. Our study is among the few that have evaluated the outcomes and survival experiences among clients who initiate ART within the first seven days of enrolment. Evidence elsewhere has shown that same-day initiation of ART after HIV diagnosis is feasible in resource-limited settings [12] and early ART initiation may yield better treatment outcomes including reduced mortality, low occurrences of opportunistic infections and early immunological recovery [6, 26]. Our study builds on this critical evidence, by demonstrating better survival experiences and time to undetectable viral load among individuals who initiated ART within seven days after enrolment, a timing that may be more feasible for individuals who require referrals from HIV diagnosis to treatment facilities.

Our findings further indicate lower rates of loss to follow-up among those who initiated early compared to those who deferred. There was a two-fold increase in the proportion lost to follow-up among those who initiated beyond seven days of enrolment; however, there could be other contributors to loss to follow-up such as death [33] and ART site [34].

Studies have shown that the baseline ART regimen [19, 20], adherence rates [27, 35] and baseline WHO clinical stage all influence time to viral suppression. Our findings show no significant contribution to undetectable viral load if a patient was started on either a Nevirapine (NVP), Efavirenz (EFV) or a protease inhibitor (PI) based regimen. Additionally findings elsewhere indicated that being initiated on a NVP-based regimen was associated with a 1.5-fold increase in undetectable viral load [20]; however in our study, there was no significant difference between patients on these regimens. This could have been influenced by the effect of treatment guidelines that recommend that all exposed infants who were exposed to NVP-based syrups be initiated on a PI-based regimen if they eventually test HIV positive amidst challenges in the use of PI based syrups [36]. However, we believe that our findings portray a realistic clinical picture amidst operational treatment challenges in health care facilities. Our findings further contribute to the body of evidence that consistently good adherence (≥95%) and WHO clinical stage at baseline influence the virological outcomes of patients [27, 35]. Findings seen in our study regarding the contribution of baseline weight on clinical improvement among HIV patients has also been shown elsewhere [37].

Despite remarkable findings in the literature on the effect of baseline viral load (>100,000 copies/ml) on virological response [22, 23], our study did not show a significant association between baseline VL and time to undetectable VL. This could be partially explained by the high proportion of paediatric patients under five years of age included in this study, as immunological response has been shown [16–18] to differ according to age category. Our study had some limitations. First this study did not ascertain the cause of death which may have included other non-HIV related causes. Secondly viral load tests were not conducted at 6 or 12 months for some patients as scheduled—this influenced the analysis of the median time to viral suppression. Despite these limitations, this study provides evidence of better treatment outcomes in individuals initiating treatment within a week.

## Conclusion

Patients newly enrolled in HIV care with WHO clinical stage I or II, who are promptly initiated on an Efavirenz-based ART regimen are more likely to achieve viral suppression. The drug regimen and consistently good adherence also plays an important role in ensuring viral suppression. Focus should therefore be targeted on the baseline regimen, timing of ART initiation, WHO clinical stage and adherence to treatment to ensure attainment of ending AIDS by 2030.

## Abbreviations

AIDS: Acquired Immunodeficiency Syndrome
ART: Antiretroviral therapy
BCM: Baylor College of Medicine, Texas
CD4: Cluster of Differentiation 4
CDC: Centers for Disease Control and Prevention
EFV: Efavirenz
EMR: Electronic Medical Record
HIV: Human immunodeficiency virus
MUAC: Mid Upper Arm Circumference
NCST: National Council for science and Technology
NVP: Nevirapine
PI: Protease inhibitor
PNC: Post Natal Care
RTI: Reverse transcriptase inhibitor
SHR: Sub-hazard ratio
VL: Viral load
WHO: World Health Organization
